# Manuals, guidelines and toolkits to support the elimination of blinding trachoma

**Published:** 2015

**Authors:** Paul Courtright, Chad MacArthur

**Affiliations:** Director: Kilimanjaro Centre for Community Ophthalmology, Cape Town, South Africa. Email: **pcourtright@kcco.net**; President: MacArthur/Tapert Global Health Consulting, South Harpswell, Maine, USA.

**Figure:**
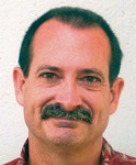
Paul Courtright

**Figure:**
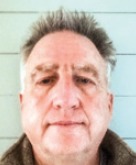
Chad MacArthur

The year 2020 is the target date for the elimination of blinding trachoma as a public health problem. There has been great progress, and there is unprecedented funding available – particularly from DFID, the Queen Elizabeth Diamond Jubilee Trust, and USAID. There is also reason for optimism that, over the next five years, further success will be seen in many endemic countries.

In order to achieve elimination, countries need the technical and programmatic capacity to implement and scale up the World Health Organizations's (WHO) recommended SAFE strategy: **S**urgery, **A**ntibiotics, **F**acial cleanliness and **E**nvironmental improvement. To assist with capacity strengthening, a number of manuals, guidelines and other tools have been developed or are in the process of being developed. Some of the materials are designated for overall programme management while others are specific to various components of SAFE. The manuals and guidelines are based on preferred practices that have been developed through analysis of the available evidence; this ensures that programmes are both effective and efficient. Those available online (see below) are referenced by number, and those with an asterisk (*) are available from the author.

Strengthening the national leadership of a trachoma programme is important in order to achieve trachoma elimination objectives. The manual ‘Guidance for Strengthening Leadership’* has therefore been developed and focus is given to strategies that delegate specific management tasks to others; this will allow the national coordinator to better lead elimination efforts. A guide for Trachoma Action Planning (TAP)^1^ has also been developed; the TAP guide assists countries to set their own ultimate intervention goals and to determine the activities needed to achieve them.

Reduction of the global backlog of trachomatous trichiasis requires focus on the quality of surgical outcomes, the quantity of trichiasis operations performed and the efficiency with which these services are provided. In response to these needs, and as an adjunct to the WHO ‘Trichiasis Surgery for Trachoma’ manual^2^, the manual ‘Training for trainers of trichiasis surgeons’^3^ has been developed to include the use of the newly-developed HEAD-START mannequin. This mannequin provides a transition for surgical trainees between classroom work and practising on a live patient

**Figure:**
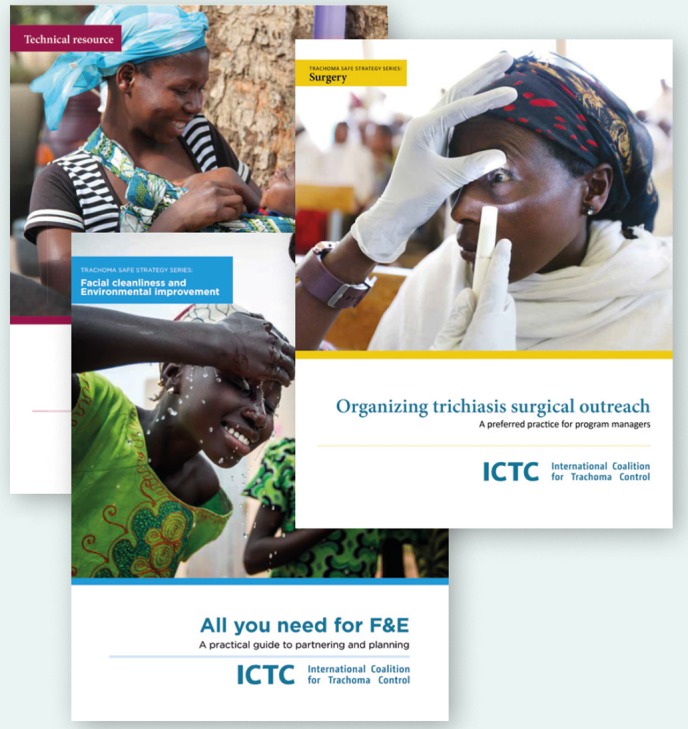
Some of the manuals available from the International Coalition for Trachoma Control

Supportive supervision of surgeons is needed to ensure the quality of trichiasis surgery and to increase productivity. A recently developed ‘Trichiasis Supervisor Training Manual’* focuses on training supervisors and providing specific supervision strategies and checklists. Two other completed manuals include: ‘Training of Trichiasis Case Finders’* and ‘Counseling of Trichiasis Patients’*. Evidence shows that most operations are conducted through the use of outreach activities; a guide for organising effective and efficient outreach campaigns has therefore been developed.^4^

Mass drug administration with Zithromax®, the antibiotic donated by Pfizer (through the International Trachoma Initiative) for trachoma prevention and elimination, seeks to bring the clinical signs of active trachoma to below elimination thresholds of less than 5% among children aged 1–9 years. A number of capacity-building materials have been developed to assist countries to achieve this, including a guide for supervision* and a training guide for distributors.* Because planning at the local level is essential for achieving the necessary antibiotic coverage, a guide for micro-planning* is also available. An additional guide is in development that aims to improve the supply chain management of Zithromax®. Last year, a handbook on managing serious adverse events^5^ was published by RTI International and is available for adaptation to specific national contexts.

Finally, work is ongoing to provide countries with support for the inclusion of F and E into their national programmes. A toolkit for F and E^6^ provides a scope of work anda set of activities for trachoma programme managers to achieve successful coordination and implementation of F and E interventions for trachoma control and elimination. The toolkit includes strategies related to ensuring coordination with the main WASH stakeholders.

Many materials can be found on the website of the International Coalition for Trachoma Control (ICTC) at **www.trachomacoalition.org**. Efforts are being made to ensure that these documents are available in French and Portuguese as well as English. A number of resources are also being translated into Arabic and Amharic. To maximise the usefulness of these tools, it is recommended that they be adapted to the context of the endemic country where they will be used.
